# Synthesis, IR Spectra, Crystal Structure and DFT Studies on 1-Acetyl-3-(4-Chlorophenyl)-5-(4-Methylphenyl)-2-Pyrazoline

**DOI:** 10.3390/molecules13092039

**Published:** 2008-09-01

**Authors:** Huan-Mei Guo

**Affiliations:** 1 Microscale Science institute, Weifang College, Weifang Shandong 261061, P. R. China; E-mail: huanmeiguo@163.com (Huan-Mei Guo); 2 Department of Chemistry, Weifang College, Weifang Shandong 261061, P. R. China; E-mail: tonglinwang@163.com (Lin-Tong Wang); 3 New Materials & Function Coordination Chemistry Laboratory, Qingdao University of Science and Technology, Qingdao Shandong 266042, P. R. China; E-mails: jing59.zhang@yahoo.com.cn (Jing-Zhang), zhaopusu@yahoo.com.cn or zhaopusu@qust.edu.cn (Pu-Su Zhao)

**Keywords:** Synthesis, Crystal structure, Vibrational frequency, DFT.

## Abstract

1-Acetyl-3-(4-chlorophenyl)-5-(4-methylphenyl)-2-pyrazoline has been synthesized and characterized by elemental analysis, IR and X-ray single crystal diffraction. Density functional (DFT) calculations have been carried out for the title compound by using the B3LYP method at the 6-311G** basis set level. The calculated results show that the predicted geometry can reproduce well the structural parameters. Predicted vibrational frequencies have been assigned and compared with experimental IR spectra and they are supported each other. On the basis of vibrational analyses, the thermodynamic properties of the title compound at different temperatures have been calculated, revealing the correlations between *C*^0^*_p, m_*, *S*^0^_m_, *H*^0^*_m_* and temperatures.

## Introduction

Fluorescent probes are powerful tools in cell biology for the non-invasive measurement of intracellular ion concentrations [1]. They have found widespread applications, for example, to gauge intracellular calcium concentrations [2], to visualize labile zinc [3,4] and iron pools [5] or as pH sensors [6]. Among various possible fluorescent probes, pyrazoline-based fluorophores stand out due to their simple structures and favorable photophysical properties such as large extinction coefficients and high quantum yields (*Ф*_f_° 0.6-0.8) [7]. Their attractive applications, including cation- or pH-sensitive probes, have been described [8,9,10], and the suitability of pyrazoline fluorophores as probes in a biological environment has also been explored [11]. Because of its modular nature, the synthesis of 1,3,5-trisubstituted pyrazoline fluorophores provides a high degree of structural flexibility [7,12]. On the other hand, density functional theory (DFT) has long been recognized as a better alternative tool in the study of organic chemical systems than the *ab initio* methods used in the past [13],since it is computationally less demanding for inclusion of electron correlation. Detailed analyses [14,15,16,17] on the performance of different DFT methods have been carried out particularly for equilibrium structure properties of molecular systems, such as geometry, dipole moment, vibrational frequency, etc. The general conclusion from these studies is that DFT methods, particularly with the use of nonlocal exchange-correlation function, can predict accurate equilibrium structure properties. With all this in mind, after the title compound of 1-acetyl-3-(4-chlorophenyl)-5-(4-methylphenyl)-2-pyrazoline was synthesized, we performed DFT calculations on it. In this paper, we wish to report the experimental values as well as the calculated results.

## Results and Discussion

### Description of the crystal structure

**Figure 1:**
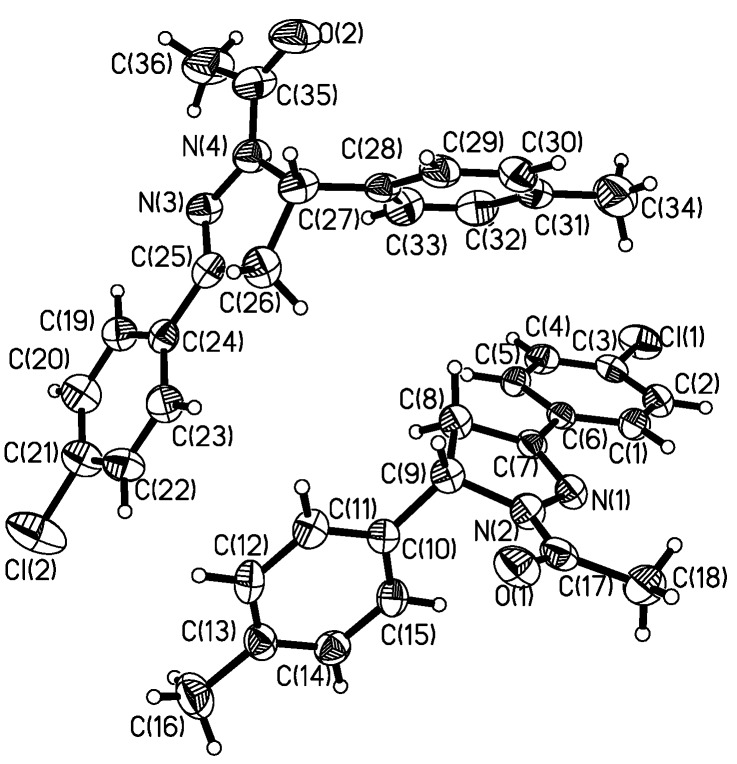
Molecular structure with the atomic numbering scheme for the title compound.

The displacement ellipsoid plot for the title compound with the numbering scheme is shown in Figure 1. Selected bond lengths and bond angles by X-ray diffraction are listed in Table 1, along with the calculated bond parameters.

**Table 1:** Selected structural parameters by X-ray and theoretical calculations.

Bond lengths (Å)	Exp.	Bond lengths	Exp.	B3LYP/6-311G**
Cl(1)-C(3)	1.745(4)	Cl(2)-C(21)	1.725(5)	1.7577
O(1)-C(17)	1.220(4)	O(2)-C(35)	1.224(5)	1.2171
N(1)-C(7)	1.293(4)	N(3)-C(25)	1.292(4)	1.2889
N(1)-N(2)	1.395(4)	N(3)-N(4)	1.398(4)	1.3699
N(2)-C(17)	1.372(5)	N(4)-C(35)	1.363(5)	1.3826
N(2)-C(9)	1.483(4)	N(4)-C(27)	1.488(5)	1.4863
C(1)-C(2)	1.382(5)	C(19)-C(20)	1.378(5)	1.3857
C(5)-C(6)	1.390(5)	C(23)-C(24)	1.395(5)	1.4018
C(6)-C(7)	1.471(5)	C(24)-C(25)	1.475(5)	1.4639
C(8)-C(9)	1.551(5)	C(26)-C(27)	1.541(5)	1.5523
C(9)-C(10)	1.515(5)	C(27)-C(28)	1.510(5)	1.5165
C(10)-C(15)	1.375(5)	C(28)-C(29)	1.374(5)	1.3933
C(10)-C(11)	1.380(5)	C(28)-C(33)	1.388(5)	1.3987
C(13)-C(16)	1.521(5)	C(31)-C(34)	1.522(6)	1.5095
C(17)-C(18)	1.502(5)	C(35)-C(36)	1.495(6)	1.513
Bond angles (°)	Exp.	Bond angles (°)	Exp.	B3LYP/6-311G**
C(7)-N(1)-N(2)	108.3(3)	C(25)-N(3)-N(4)	107.4(3)	109.3937
N(1)-N(2)-C(9)	113.3(3)	N(3)-N(4)-C(27)	113.3(3)	113.5694
N(1)-C(7)-C(8)	113.8(3)	N(3)-C(25)-C(26)	114.7(4)	113.0852
C(7)-C(8)-C(9)	103.0(3)	C(25)-C(26)-C(27)	102.8(3)	102.7091
N(2)-C(9)-C(8)	100.9(3)	N(4)-C(27)-C(26)	101.1(3)	100.7838
C(17)-N(2)-N(1)	122.9(3)	C(35)-N(4)-N(3)	122.8(4)	122.7854
C(2)-C(1)-C(6)	121.4(4)	C(20)-C(19)-C(24)	120.6(4)	120.984
C(3)-C(4)-C(5)	119.1(4)	C(23)-C(22)-C(21)	120.2(4)	119.1608
C(1)-C(6)-C(7)	121.1(4)	C(19)-C(24)-C(25)	121.0(4)	120.9516
C(15)-C(10)-C(11)	117.2(3)	C(29)-C(28)-C(33)	118.3(4)	118.401
C(13)-C(14)-C(15)	121.8(4)	C(31)-C(32)-C(33)	121.6(4)	121.1049
C(12)-C(13)-C(16)	121.1(4)	C(32)-C(31)-C(34)	121.8(5)	120.8844
O(1)-C(17)-N(2)	119.5(4)	O(2)-C(35)-N(4)	119.3(5)	119.787
O(1)-C(17)-C(18)	124.4(4)	O(2)-C(35)-C(36)	124.0(5)	123.9266
N(2)-C(17)-C(18)	116.1(4)	N(4)-C(35)-C(36)	116.8(4)	116.2863

The structure of the title compound contains two crystallographically independent molecules in the asymmetric unit, hereafter named S1 [containing C(1)~C(6) phenyl ring] and S2 [containing C(19)~C(24) phenyl ring]. In S1 and S2, all of the bond lengths and bond angles are different. For example, C-Cl bond length of 1.745(4) Å in S1 is longer than that in S2 (1.725(5) Å). In S1, all the bond lengths in two phenyl rings are in the 1.375(5) ~ 1.390(5) Å range, while in S2, all the bond lengths in two phenyl rings fall within the 1.374(5) ~ 1.395(5) Å range. Despite some differences, all of the bond lengths and bond angles in the phenyl rings are in the normal range. As for the two pyrazolinyl rings, the bond lengths of C=N[1.293(4) Å], C-N [1.483(4) Å ] and N-N [1.395(4) Å] in S1 are all comparable with those of C=N[1.292(4) Å], C-N [1.488(5) Å ] and N-N [1.398(4) Å] in S2, and they are all corresponding to those found in similar structures[11,18], respectively. The bond angles in the two pyrazolinyl rings are also in good agreement with those in the above cited structures [11,18]. The dihedral angles between the pyrazolinyl ring with the phenyl rings at positions 3 and 5 of the pyrazoline are 14.00(2) and 83.84(3)° in S1 and 3.54(2) and 78.46(2)^o^ in S2, respectively. 

In the crystal lattice, there are two potentially weak intramolecular interactions, along with one intermolecular interaction (C-H···Y, Y=O, N) [19]. For the two intramolecular interactions, the distances and angles between donor and acceptor are 2.7991(2) Å and 100.44(2)° for C(18)-H(18B)···N(1) and 2.7989(2) Å and 108.47(2)° for C(36)-H(36B)···N(3), respectively. For the intermolecular interaction, the distance and angle between donor and acceptor is 3.4655(2) Å and 166.12(2)° for C(5)-H(5)···O(1) [symmetry code: *x*, 3/2-*y*, -1/2+*z*]. In the solid state, the above supramolecular interactions stabilize the crystal structure.

### Optimized geometry

**Figure 2:**
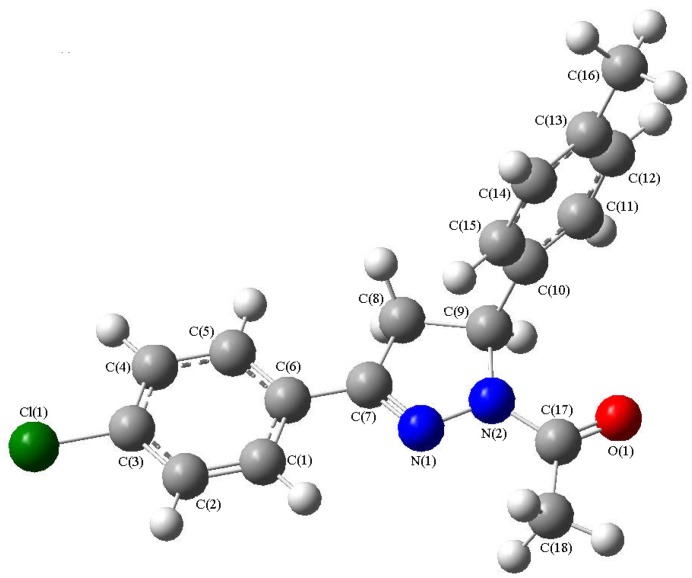
One optimized molecular structure for the title compound.

Although there are two independent molecules in the asymmetric unit and these two molecules have some different bond lengths and bond angles in the solid state, they denote the same compound. So, only one molecular structure was selected to be optimized in the gas phase. DFT calculations were performed at B3LYP/6-311G** level of theory and the optimized structure was shown in Figure 2. Some optimized geometric parameters are also listed in Table 1. Comparing the theoretical values with the experimental ones indicates that most of the optimized bond lengths are slightly larger than the experimental values, as the theoretical calculations are performed for isolated a molecule in gaseous phase and the experimental results are for a molecule in a solid state.

The geometry of the solid-state structure is subject to intermolecular forces, such as van der Waals interactions and crystal packing forces. The biggest differences of bond lengths and bond angles between the experimental and the predicted values are -0.0281 Å for Cl(2)-C(21) bond distance and 1.9937° for C(25)-N(3)-N(4) bond angle, which suggests that the calculational precision is satisfactory [20] and the B3LYP/6-311G** level of theory is suitable for the system studied here. Based on the optimized geometries, IR spectra and thermodynamic properties of the title compound are discussed as follows.

### Vibrational frequency

**Figure 3:**
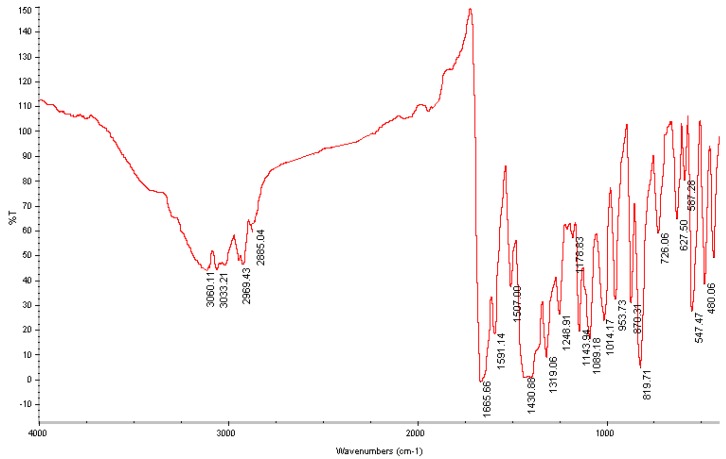
Experimental IR spectrum of the title compound.

The experimental IR spectrum is shown in Figure 3. Vibrational frequencies calculated at the B3LYP/6- 311G** level were scaled by the typical factor 0.96. Some primary calculated harmonic frequencies are listed in Table 2 and compared with the experimental data. The descriptions concerning the assignment have also been indicated in the Table 2. The Gauss-view program [21] was used to assign the calculated harmonic frequencies. As seen from Table 2, the predicted harmonic vibration frequencies and the experimental data are very similar to each other. The biggest error occurs at C-H stretching vibration, with the biggest deviation being 36 cm^-1^. In a word, the scaled frequencies of the DFT calculation are close to the corresponding FT-IR vibration data and on the whole the DFT-B3LYP/6-311G** level can predict the vibrational frequencies for the system studied here.

**Table 2:** Comparison of the observed and calculated vibrational spectra of the title compound.

Assignments	Exp. IR (with KBr)	Calculated ( B3LYP/6-311G** )
phenyl ring C-H str.	3066	3080-3030
acetyl C-H str.	3033	3026
pyrazolinyl ring C-H str.	2969	2966
methyl group C-H str.	2885	2901
C=O str.	1666	1681
phenyl ring C=C str.+ C=N str.	1591	1591-1577
phenyl ring C=C str.	1507	1486
methyl group C-H bend	1430	1437
phenyl ring C-H bend + pyrazolinyl ring C-H bend	1319	1328
pyrazolinyl ring C-H bend + N-N str.	1248	1248
pyrazolinyl ring C-H bend + N-N str.	1144	1138
pyrazolinyl ring C-H bend	1089	1088
methyl group C-H bend	1014	1019-1011
phenyl ring C-H bend	953	950
phenyl ring C-H twist.	819	815
skeleton deformation + C-Cl str.	726	715
skeleton deformation	627	630

### Thermodynamic properties

On the basis of vibrational analyses and statistical thermodynamics, the standard thermodynamic functions: heat capacity (*C*^0^*_p, m_*), entropy（*S*^0^*_m_*）and enthalpy（*H*^0^*_m_*）were obtained and listed in Table 3. The scale factor used for frequencies was also 0.96. As observed from Table 3, the values of *C*^0^*_p,m_*,*S*^0^*_m_* and *H*^0^*_m_* all increase with the increase of temperature from 100.0 to 800.0 K, which is attributed to the enhancement of the molecular vibration while the temperature increases. 

**Table 3:** Thermodynamic properties at different temperatures at B3LYP/6-311G** level.

*T* (K)	*C*^0^*_p,m_* (J·mol^-1^·K^-1^)	*S*^0^*_m_* (J·mol^-1^·K^-1^)	*H*^0^*_m_* (kJ·mol^-1^)
100.0	147.83	422.98	9.79
200.0	239.08	552.87	29.05
298.1	337.74	666.57	57.32
300.0	339.61	668.66	57.95
400.0	435.62	779.80	96.81
500.0	516.58	886.01	144.56
600.0	581.88	986.18	199.60
700.0	634.50	1079.97	260.51
800.0	677.47	1167.59	326.18

The correlation equations between these thermodynamic properties and temperature *T* are as follows: 

*C*^0^*_p,m_* = 20.523 + 1.235 *T* - 5.111*10^-4^*T ^2^* ( *R*^2^ = 0.9989)

*S*^0^*_m_* = 293.849 + 1.343 *T* - 3.141*10^-4^*T ^2^* ( *R*^2 ^= 0.99997 )

*H*^0^_m_ =-6.360 + 0.1010 *T* + 3.970*10^-4^*T ^2^*( *R*^2 ^= 0.9997 )

These equations will be useful for the further studies on the title compound.

## Experimental

### General

IR spectra (4000-400 cm^−1^), were recorded as KBr pellets on a Nicolet FT-IR spectrophotometer at room temperature. 

### Synthesis

All chemicals were obtained from a commercial source and used without further purification.

1-(4-Chlorophenyl)-3-(4-methylphenyl)-2-propenyl-1-ketone (0.01 mol) and hydrazine hydrate (0.015 mol) were mixed in acetic acid (40 mL) and stirred in refluxing for 6 h, then the mixture was poured into ice-water to afford yellow solids. The solids were filtrated and washed with water until the pH of solution was about 7. Finally, the yellow solid crystals were dry under room temperature. Yield 89 %. m.p. 123-125^o^C. Found: C, 69.04; H, 5.42; N, 5.03 %. Calc. for C_36_H_34_Cl_2_N_4_O_2_: C, 69.11; H, 5.48; N, 5.12 %.

### Theoretical methods

DFT calculations with a hybrid functional B3LYP (Becke’s three parameter hybrid functional using the LYP correlation functional) at basis set 6-31G* by the Berny method [22] were performed with the Gaussian 03 software package [23]. Vibrational frequencies calculated ascertain the structure was stable (no imaginary frequencies). The thermodynamic properties of the title compound at different temperatures were calculated on the basis of vibrational analyses. All calculations were performed on a DELL PE 2850 server and a Pentium IV computer using the default convergence criteria.

### Crystal structure determination

The diffraction data were collected on a Enraf-Nonius CAD-4 diffractometer with graphite-monchromated Mo-K*α* radiation (*λ* = 0.71073 Å, *T* = 293K). The technique used was *ω*-2*θ* scan mode with limits 1.94 to 25.02 º. The structure of the title compound was solved by direct methods and refined by least squares on *F*^2^ by using the *SHELXTL* [24] software package. All non-hydrogen atoms were anisotropically refined. The hydrogen atom positions were fixed geometrically at calculated distances and allowed to ride on the parent carbon atoms. The molecular graphics were plotted using *SHELXTL*. Atomic scattering factors and anomalous dispersion corrections were taken from [25]. A summary of the key crystallographic information is given in Table 4. CCDC 696833 contains the supplementary crystallographic data for this paper. These data can be obtained free of charge via www.ccdc.cam.ac.uk/conts/retrieving.html (or from the CCDC, 12 Union Road, Cambridge CB2 1EZ, UK; fax: +44 1223 336033; e-mail: deposit@ccdc.cam.ac.uk)

**Table 4:** Crystal data and structure refinement.

Empirical formula	C_36_H_34_Cl_2 _N_4_O_2_
Formula weight	625.57
Temperature	293(2) K
Wavelength	0.71073 Å
Crystal system, space group	Monoclinic, P2(1)/c
Unit cell dimensions	*a* = 18.158(16) Å
	*b* = 13.461(12) Å *β*= 112.654(16) ^o^
	*c* = 14.751(14) Å
Volume	3327(5) Å^3^
*Z*, Calculated density	4, 1.249 Mg/m^3^
Absorption coefficient	0.233
*F*(000)	1312
*θ* range for data collection	1.94 to 25.02 °
Limiting indices	-21 ≤ *h* ≤ 19, -13 ≤ *k* ≤ 16, -17 ≤ *l* ≤15
Reflections collected / unique	16668 / 5851 [*R*_in t_= 0.0835]
Refinement method	Full-matrix least-squares on *F*^2^
Data / restraints / parameters	5851 / 18 / 401
Goodness-of-fit on *F*^2^	1.001
Final *R* indices [*I*>2*σ*(*I*)]	*R*_1_ = 0.0500, *wR*_2_ = 0.1123
*R* indices (all data)	*R*_1_ = 0.1730, *wR*_2_ = 0.1586
Largest diff. peak and hole	0.180 and -0.227 e. Å^-3^
